# Management of a Traumatic Collapse of the First Carpometacarpal Joint After Trapeziectomy With Suture Suspensionplasty: A Case Report

**DOI:** 10.7759/cureus.60216

**Published:** 2024-05-13

**Authors:** Landan R Ruff, Blake E Delgadillo, George F El-Bahri

**Affiliations:** 1 Orthopedic Surgery, Lake Erie College of Osteopathic Medicine, Bradenton, USA; 2 Orthopedic Surgery, Bahri Orthopedics and Sports Medicine Clinic, Jacksonville, USA

**Keywords:** traumatic arthroplasty failure, suture button suspensionplasty, thumb carpometacarpal arthritis, revision arthroplasty, thumb cmc arthroplasty

## Abstract

Arthritis of the first carpometacarpal (CMC) joint is a common pathology hand surgeons encounter. Treatment begins with conservative measures, but when they fail, surgery is a viable option for providing relief to patients. The most widely used surgical technique is CMC arthroplasty with ligament reconstruction and tendon interposition (LRTI). However, more novel techniques such as trapeziectomy with suspensionplasty are gaining popularity. When surgical measures fail, it is important to identify the mechanism of failure and proper treatment options. There are multiple options for revision surgery at the surgeon’s disposal, with no consensus on a superior technique. This case illustrates a patient with painful subsidence secondary to a traumatic collapse of the first CMC joint eight months status post suspensionplasty with trapeziectomy. After conservative measures failed to provide relief, it was decided that a surgical revision was appropriate. The surgeon chose to move forward with suture button suspensionplasty, as it has multiple advantages over LRTI. In the short-term follow-up after revision, the patient experienced improvements in pain and range of motion, along with radiographic evidence of proper alignment of the first metacarpal without subsidence. Regarding the treatment of a case such as this, the authors believe this case should serve as a reference that may be used by future physicians when deciding which surgical technique to employ for the revision of a traumatically collapsed first CMC joint after trapeziectomy with CMC joint suspensionplasty.

## Introduction

The first carpometacarpal (CMC) joint is a biconvex saddle-shaped ball-and-socket joint formed by the first metacarpal and trapezium [1,2]. The shape of the joint allows for greater mobility while sacrificing stability. In women, a smaller, shallow, and more incongruent joint with less articular cartilage is seen, which puts them at a greater risk of osteoarthritis (OA) of the first CMC joint [1,3]. Radiological changes have an estimated 30% prevalence in post-menopausal women, but most remain asymptomatic [4]. Although OA of the distal interphalangeal joints is the most common arthritis of the hand, first CMC joint arthritis has an estimated prevalence of 10%, which makes it the second most common [5,6].

Diagnosis typically consists of a complete history, physical exam, and wrist radiographs. The important aspects of the history include age, sex, handedness, and occupation. Physical exam often consists of assessing the presence of crepitus, tenderness to palpation, range of motion and stability testing, and the grind test [5]. With a specificity of 80-93% and sensitivity of 42-53%, the grind test is a provocative maneuver that consists of applying an axial load while grinding the thumb metacarpal against the trapezium. A positive test is determined by the presence of pain with or without crepitus of the joint [7]. The Eaton classification is used as the standard radiological assessment for staging of the CMC joint OA [7,8].

Non-surgical management, which includes rest, splinting, non-steroidal anti-inflammatory drugs (NSAIDs), cortisone injections, and physical therapy for three months, is considered the first-line therapy for CMC joint arthritis [1,4-6], although surgical reconstruction is indicated when non-surgical treatments fail. Using the history completed earlier in each patient's course of treatment, the type of surgical management is dependent on age, functional demands, and stage of the disease. The choices of treatment include ligament reconstruction, metacarpal extension osteotomy, trapezial resection, interpositional arthroplasty, ligament reconstruction and tendon interposition (LRTI), partial trapeziectomy, prosthetic CMC arthroplasty, resurfacing arthroplasty, interposition arthroplasty, total joint replacement arthroplasty, and arthrodesis [1,4,5]. Newer techniques include suspensionplasty with trapeziectomy [9]. Surgical treatment yields an 80-90% patient satisfaction rate, but no matter the surgical intervention selected, every patient is at risk of CMC joint hyperextension and collapse deformity [4]. Additionally, for both volar and dorsal approaches, the proximity of palmar cutaneous branch of the median nerve and dorsal sensory branch of the radial nerve put them at risk of injury (laceration or traction) with a possible resultant complex regional pain syndrome (reflex sympathetic dystrophy) or neuroma formation [4,5].

The purpose of this study is to explore the management of a traumatically torn thumb implant after trapeziectomy with suture suspensionplasty for the treatment of first CMC joint osteoarthritis.

## Case presentation

A 56-year-old female who is right-hand dominant presented to the clinic with right first CMC joint and first digit pain, which had been ongoing for several years. The patient reported the pain had gradually worsened over the past year, which she attributed to overuse. She neither had previous trauma nor any prior surgeries on the digit. The pain was described as constant, aching, and throbbing, which limited her activities of daily living that involved the need to grip items. In the past, she had previously attempted to mitigate the pain with topical diclofenac sodium, bracing, and cortisone injections with only mild symptomatic relief. Due to limited improvement with prior conservative treatment, other treatment modalities were discussed, including surgery.

**Figure 1:**
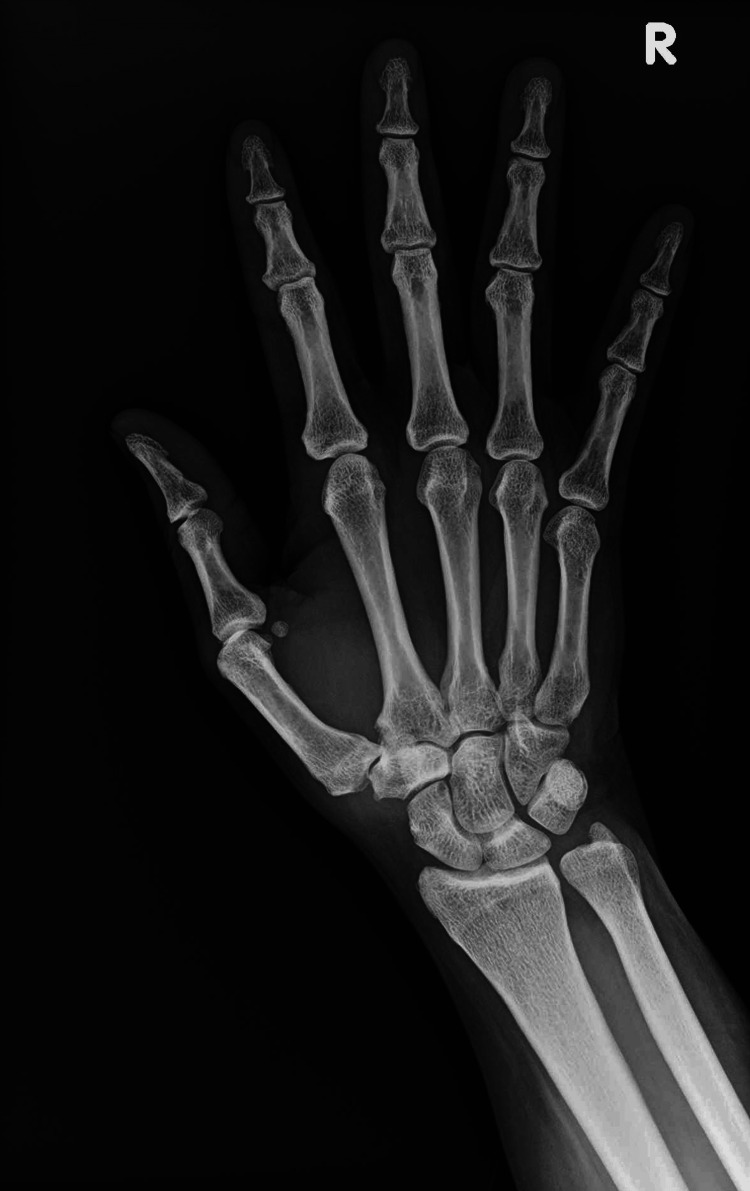
Posteroanterior radiograph of the right hand demonstrating moderate osteoarthritis of the first carpometacarpal joint as evidenced by decreased joint space, osteophytes, and subluxation.

Physical exam revealed a positive grind test and tenderness to palpation of the joint. A full range of motion was noted, and all compartments of the hand were soft. The neurovasculature of the right hand was found to be fully intact. Plain posteroanterior (PA) radiograph of the right hand showed moderate OA of the first CMC joint (Figure 1). After a thorough discussion with the patient, the decision to proceed with surgical intervention was made.

**Figure 2:**
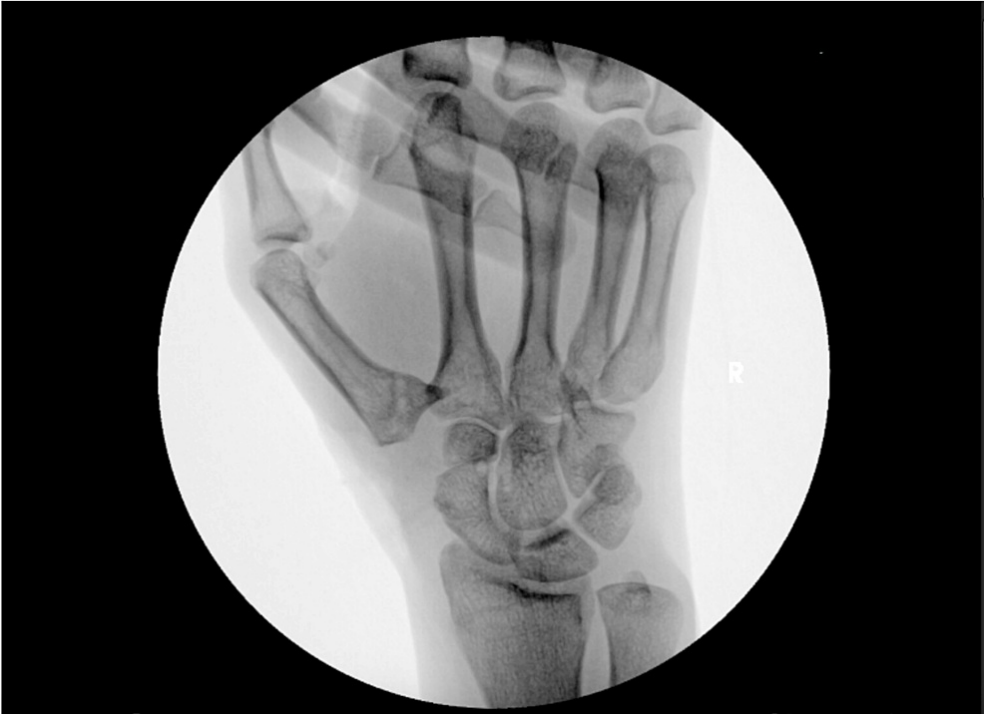
Intraoperative posteroanterior radiograph of the right hand demonstrating the absence of the trapezium after trapeziectomy with proper alignment of the first metacarpal.

When it was time for the surgical procedure in February 2023, the patient was sterilely prepped and draped, and surgery was begun. The plan was to employ a right-hand trapeziectomy with first CMC joint suture suspensionplasty using Arthrex FiberLock, Arthrex 2.9 PushLock, and Arthrex OrthoFLEX graft. The standard dorsal approach to the first CMC joint was used, and the skin and capsule were dissected through, while the neurovascular and tendinous structures were protected. The trapezium was then removed, which was confirmed with C-arm fluoroscopy. A hole was drilled into the second metacarpal. The FiberLock was then inserted, which showed excellent fixation. A hole was drilled into the first metacarpal, and the internal brace from the FiberLock was inserted into a 2.7 screw. Upon inserting the screw into the first metacarpal, the screw broke off. This was repeated, yielding the same outcome. The decision was then made to use a PushLock, which was inserted into the first metacarpal with sutures. Extensive fixation was achieved. The Arthrex OrthoFLEX graft was then cut into four pieces. A fourth of it was involved with the sutures, and then it was placed into the empty joint space. C-arm fluoroscopy was used to confirm the proper position of the first metacarpal (Figure 2). The CMC joint capsule and skin were closed in the standard fashion, and a thumb spica splint was applied. Overall, the procedure was carried out in the technique described by Arthrex for CMC joint suspensionplasty with the FiberLock^TM^ Suspension System. As an exception, a PushLock was used instead of a SwiveLock due to the aforementioned screw failure.

On postoperative day (POD) 3, the patient reported swelling and moderate pain, which was tolerated with pain medication. A new thumb spica splint was applied. The patient was then instructed to begin physical therapy (PT) three times per week for four weeks. On POD 28, the patient reported improvement in swelling and pain. She had nearly regained the full range of motion of her thumb, although there was an inability to run her thumb down her fifth digit. She was tolerating PT well, and she was told to continue her current PT regimen wearing the thumb spica splint for four additional weeks. At the eight-week postoperative visit, the patient regained full thumb range of motion, but she still was experiencing mild stiffness. At this point, she was advised to do home exercises and discontinue the thumb spica splint. The stiffness had improved by the twelve-week postoperative appointment. As a result, there was no need for the patient to return to the clinic unless she experienced pain or decreased range of motion in her right thumb.

**Figure 3:**
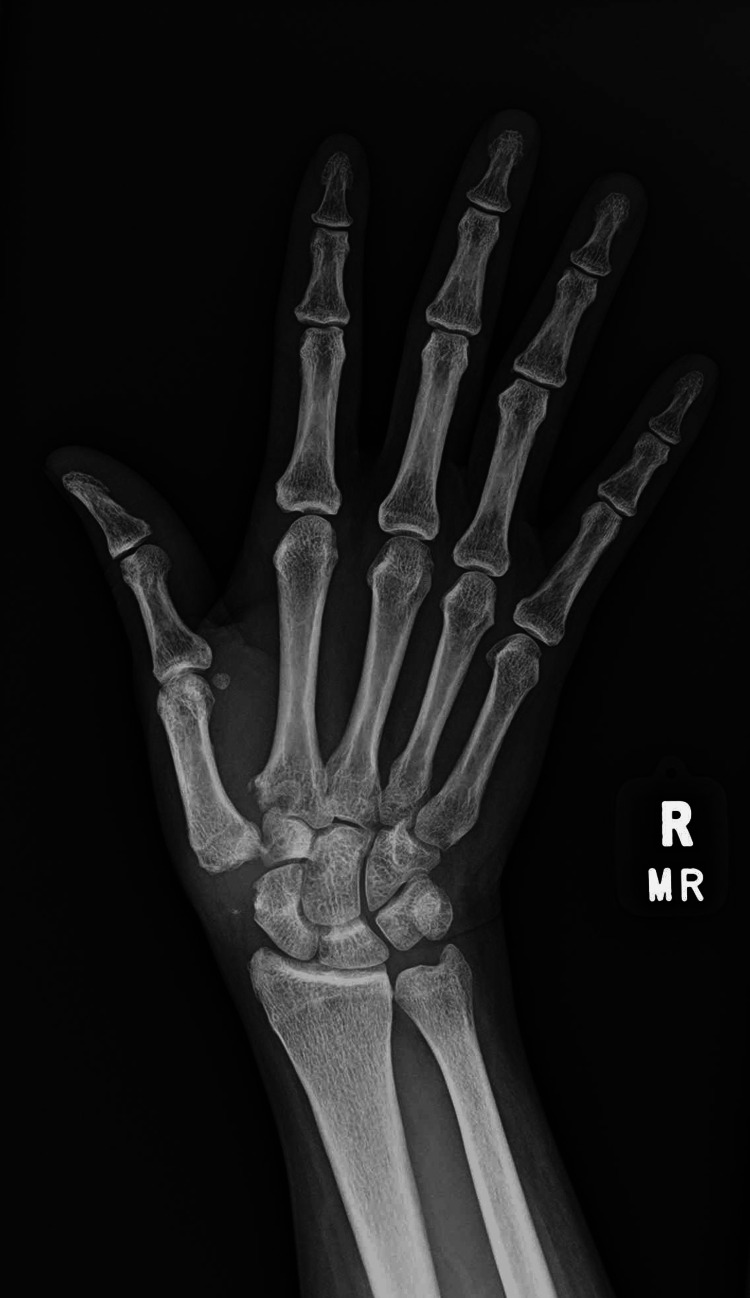
Posteroanterior radiograph of the right hand demonstrating a complete collapse of the first carpometacarpal joint. There is marked subsidence of the first metacarpal.

Eight months after her initial surgery, the patient returned to the clinic after falling on an outstretched hand the previous month. She complained of swelling, tightness, and pain in the same region she had surgery, which had worsened over the past week. A physical exam revealed a limited and painful range of motion of the right first digit with mild swelling. A PA radiograph showed a complete collapse of the right first CMC joint, indicating a torn right thumb implant (Figure 3). In an attempt to employ conservative measures to decrease pain, she was instructed to remain in the thumb spica splint to immobilize the joint until her next follow-up appointment.

At the one-week (eight months and one week post-op) and four-week (nine months post-op) follow-up appointments, the patient was still experiencing swelling, tightness, and painful range of motion in her right thumb despite the use of a thumb spica splint and at-home gentle range of motion exercises. Physical exam showed persistent swelling with a limited and painful range of motion of the right thumb. At that time, it was decided that surgical revision of the suspensionplasty would best benefit the patient.

**Figure 4:**
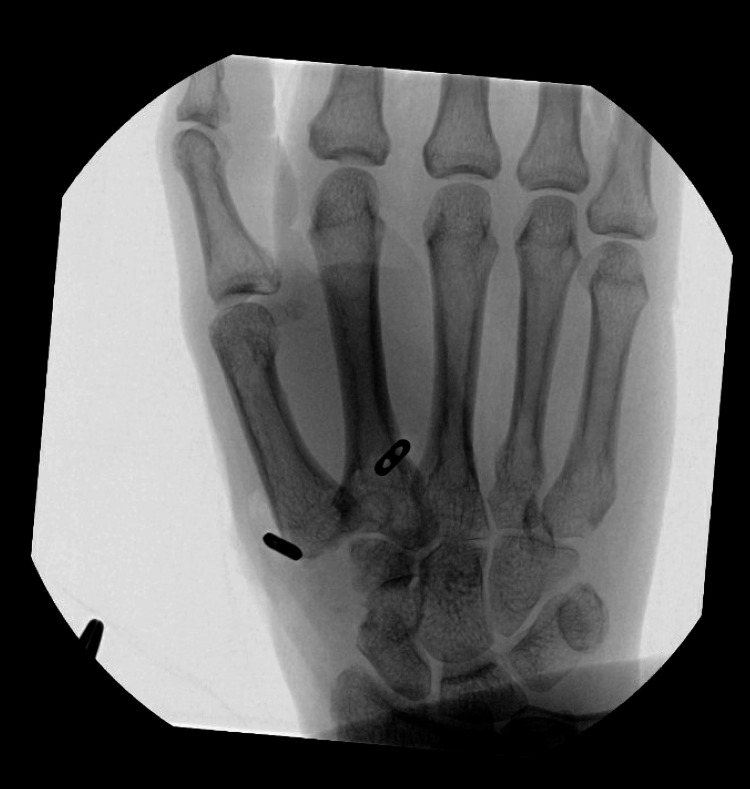
Intraoperative posteroanterior radiograph of the right hand demonstrating proper alignment of the first metacarpal. The hardware is intact and positioned properly.

In December 2023, the patient was prepped and draped in the normal fashion, and the standard dorsal approach was used. She then underwent a right-first CMC joint suspensionplasty revision using Arthrex Mini TightRope. After dissecting down to the base of the first metacarpal, the broken suture was retrieved, which was found to be torn from the second metacarpal without bony avulsion. A second incision was made over the dorsal aspect of the second metacarpal. A careful dissection was performed down to the second metacarpal, with the identification and protection of a branch of the dorsal radial sensory nerve. A hole was then drilled through the base of the first metacarpal and into the second metacarpal under fluoroscopy. A K-wire was used to pass the Mini TightRope FiberWire completely through the second metacarpal. The suture was pulled until the button came in contact with the radial side of the first metacarpal. A second button was loaded onto the suture on the ulnar side of the second metacarpal. Traction and adduction were applied on the thumb while the button was fixated onto the second metacarpal. Fluoroscopy was used to ensure proper tension of the suture and excellent position of the first metacarpal (Figure 4). The second dorsal interosseous fascia, CMC joint capsule, and skin were closed in the standard fashion, and the patient was then placed in a thumb spica splint.

**Figure 5:**
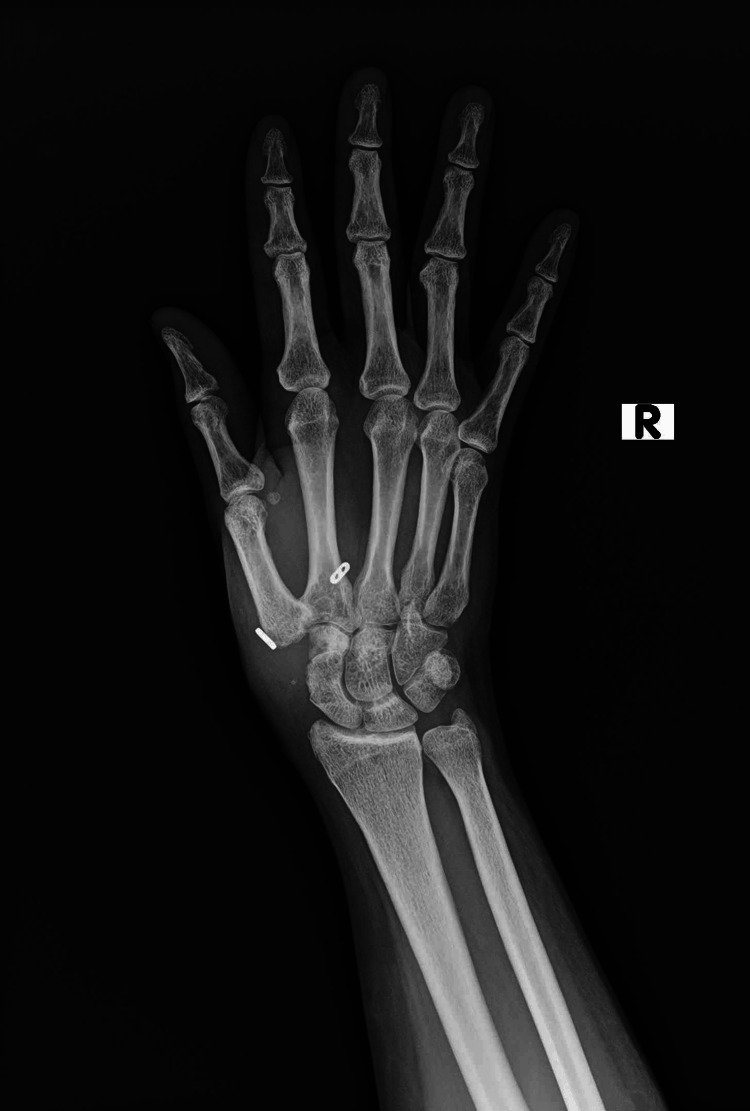
Posteroanterior radiograph of the right hand at one week post first carpometacarpal joint arthroplasty revision surgery with suture button suspensionplasty. Maintenance of the metacarpal suspension with a proper alignment of the first metacarpal can be seen.

On POD 7 (after revision surgery), the patient was doing well with only mild pain. A PA radiograph of the right hand revealed intact hardware with proper alignment of the first metacarpal (Figure 5). The thumb spica splint was removed and the patient was placed in a thumb spica orthosis for the following four weeks. The patient declined a PT referral at this visit, so it was decided that she begin a home exercise program. Instructions for home exercises were provided to the patient, which she was advised to perform daily. On POD 28, the patient reported improvement in both pain and range of motion of her right thumb. The patient was instructed to continue her home exercise program and remain in the thumb spica orthosis for an additional two weeks.

## Discussion

Although the literature regarding a traumatic first metacarpal collapse following trapeziectomy with suture suspensionplasty is scarce, other complications have been well documented. These include paresthesias and scar tenderness, injury to the dorsal branch of the radial artery, impingement between the thumb and index metacarpal bones, proximal thumb metacarpal subsidence, and suture anchor failure [10]. One study mentioned a case of pain after a traumatic fall after suture suspensionplasty, but this study excluded patients requiring revision surgery; therefore, neither the extent of the injury nor the outcome after the fall could be determined [11]. Weber et al. reported a traumatic first metacarpal dislocation from a fall after surgical intervention for first CMC joint arthritis [12]. However, this patient underwent trapeziectomy with LRTI as opposed to suture suspensionplasty performed on the patient in this study. Furthermore, the revision surgeries were different between the patient discussed in this article and the patient in the aforementioned case.

When considering the need for revision surgery, it is vital to understand the underlying etiology. It is the general consensus that neurogenic causes of pain (tender surgical scar, neuroma, or complex regional pain syndrome) should be treated first with conservative measures with early desensitization therapy and pain management intervention. However, mechanical causes of CMC joint arthroplasty failure is a prime indication for surgical revision. These causes of failure can occur after implant arthroplasty (due to implant loosening or breakage), arthrodesis (due to failure to achieve solid fusion), or suspensionplasty with or without LRTI (due to painful bony impingement secondary to subsidence) [13]. Johnson et al. reports that revision surgery rates for CMC joint arthroplasty are estimated to be 2.6-4.6% with the most common reason being painful subsidence [14], which is the main focus of this study. 

As discussed in the literature, options for revision due to painful subsidence, be it because of trauma or occur organically, include LRTI (as originally described by Burton and Pellegrini [15]), suture button suspensionplasty (SBS) with Arthrex Mini TightRope [16], and distraction pinning and soft tissue interposition [17]. Although a superior surgical technique for revision is yet to be identified by previous research, studies have shown LRTI [18] and distraction pinning and soft tissue interposition yield satisfactory results [17]. However, these surgeries require rigid immobilization for four to six weeks before the range of motion exercises can be initiated. Guerrero et al. compared LRTI to SBS and concluded SBS is an effective option for the surgical revision of CMC arthritis [19]. SBS has a shorter intraoperative, return-to-work, and immobilization time when compared to LRTI [11]. In cadaveric models, SBS showed greater resistance to immediate loading than LRTI with regard to subsidence [20]. Since the most likely reason for suspensionplasty failure in the patient presented in this case was axial loading of the first digit, the surgeon decided SBS would be the best revision surgery for long-term success. 

When identifying candidates for revision surgery and the surgical technique employed, a careful consideration needs to be made. There are multiple options for revision surgery, and the lack of agreement for which technique is superior requires approaching each patient on a case-by-case basis [13]. In the short-term follow-up period after revision with SBS in this study, the patient reported improvements in pain and range of motion along with radiographic evidence of proper alignment of the first metacarpal, as discussed previously (Figure 5).

Although SBS has been well documented as an effective surgical intervention for OA of the first CMC, there is a lack of literature for the revision process. The purpose of this case study was to highlight a relatively novel surgical revision option for a patient who sustained a traumatic collapse of the first CMC joint after trapeziectomy with CMC joint suspensionplasty. The authors believe this study calls attention to the importance of choosing the proper revision technique while additionally exhibiting the efficacy of SBS after a traumatic collapse of the first CMC joint originally treated with trapeziectomy with suture suspensionplasty. Due to the lack of literature regarding cases pertaining to this problem, the authors encourage future research to focus on quantifying the efficacy of the aforementioned revision surgery.

## Conclusions

The case presented in this manuscript highlights the efficacious use of suture button suspensionplasty after a traumatic collapse of a first carpometacarpal joint originally treated with trapeziectomy with carpometacarpal joint suspensionplasty, which is a relatively novel surgical technique. The decision on how to treat such an injury has not been universally accepted, perhaps due to lack of research in this area. Regarding the treatment of such a case, the authors believe this case should serve as a data point that may be used by future physicians when deciding which surgical technique to employ for the revision of a traumatically collapsed first carpometacarpal joint after trapeziectomy with carpometacarpal joint suspensionplasty.
